# 

**Published:** 1858-01

**Authors:** 


### INDEX TO VOL. I.

PAGE.

Address before the Esculapian Society, by E. Read, M.D. 5
Allport, W. W., D. D. S., on Diseases of the Teeth 53
American Medical Association, 96, 186, 230, 266, 340
Allaire, P. A., on Polypiform Concretions in the Heart 117
Annual Commencement of Rush Medical College 142
Anniversary Discourse before the N.Y. Academy of Medicine 174
Angel, L. H., Cases in Practice 258
Apology and Bills 414
Anchylosis of both Elbow Joints, by J. W. Freer, M.D., etc. 509
Appointments, Professorial 544
Address, Introductory to the 16th Annual College Term 562
Aurora City Medical Association, Proceedings of 577
Aneurism, Treatment of, by Compression 598
Advancement of Medical Science, a Public Address on, by O. Q. Herrick, M.D. 613

Bailey, F. K., Report on Practical Medicine 519
Belmont Medical Journal 535
Brainard, Daniel, on Ununited Fractures 421
Brainard, Daniel, on Resection of the Elbow 435
Byford, W. H., on Hepatic Abscess 439
Bucknill and Tuke on Insanity 464
Byford, W. H., on Therapeutics of Muscular Exercise 357
Byford, W. H., Translations 396
Brain, Disease of, by B. Woodward, M.D. 391
Brainard, Daniel, on Resection of the Knee Joint 293
Brown, J. H., on Medical Education 306
Brackett, C., Surgical Cases 245
Benson, J. W., on Asphyxia and Hysteria from Chloroform 210
Bevan, Thomas, Translations by 216, 224
Bevan Thomas, Cases in Obstetrical Practice. 32
Bailey, F. K., on Erysipelas Neanatorum 161
Byford, W. H., Translations of 36, 38, 41, 171, 170, 167
Byford, W. H., Case of Dr. Bacon 127
Bacon, Milton, Death of 127
Brackett, Charles, Cases in Practice 76
Byford, W. H., on Prone Position in Examining Fœtal Circulation 77
Brower, J. H., Two Cases of Ovarian Dropsy Cured 212

College Clinic, Cases treated by Prof. Brainard 28
Cases in Obstetrical Practice, by Dr. Bevan 32
Correction 48
Correspondents 190, 94, 50
Cases in Practice, by Dr. Brackett 76
Crooks, J. W., on Enlargement of the Spleen 123
Children, Diseases of, by J. Forsyth Meigs 132
Cases in Practice, by E. Read 149

PAGE.

Collodion for Undeveloped Nipples 170
Changes, Editorial 178
Cancer of the Pylorus, by R. N. Isham, M.D. 204
Casselberry, Isaac, on the Use of Iron 225
Campbell, R., on Dysentery 229
Cases in Practice, by L. H. Angel, M.D. 258
Carnochan, J. M., on Operative Surgery and Pathology 263
Change in the General Treatment of Diseases, by J. M. Hinkle, M.D. 311
Chicago, its Sanitary Condition, by N. S. Davis 402, 342
Chambers, W. M., on Diseases of the Cervix and Os Uteri 451
Convulsions, Puerpural, by R. N. Isham, M. D. 485
Cases in Practice, by Thos. W. Fry, M.D. 503
Clinic at the Mercy Hospital, by N. S. Davis, M. D. 538
Cook Co. Medical Society, Annual Meeting of 190
Close of the Volume 662
Croup, Cold Applications and Sulph. of Copper in 667
Chloroform, Death from 667

Delirium Tremens, treated by Ipecacuanha 576
Dickinson, William, on Strangulated Hernia 497
Dowler, Bennett, on Preserving Bodies for Dissection 264
DeBruler, J. P., on Milk-Sickness 206
DeBruler, J. P., on Air Entering the Veins During Labor 114
Diseases of the Teeth, by W. W. Allport, D. D.S. 53
Diagnostic Sign of Pregnancy, by W. H. Byford, M.D. 77
DeWitt Co. Medical Society 79
Death from Chloroform 667
Diabetes, by W. Matthews, M.D. 635

Ethics, Vexed Questions in 88
Empyema, treated by Injections, by David Prince, M.D. 95
Education, Medical 136
Erysipelas Neanatorum, by Dr. Bailey 161
Epilepsy and Epileptiform Convulsions 216
Esculapian Society, Semi-Annual Meeting of 326
Elements of Inorganic Chemistry 334
Extraction of Bones of a Fœtus, by D. W. Young, M.D. 383
Editorial 472
Enquiry Answered 606

Freer, J. W., Introductory Address 562
Freer, J. W., Anchylosis of Elbow Joints 509
Freer, J. W., on a New Mode of Dressing Fractured Clavicle 517
Fever, Bilious, with Torpor of Brain, by Drs. Stone and Dewey 573
Fry, Thos. W., Cases in Practice 503
Fisher, A., Case of Inversion of the Uterus 510
Fractures of the Lower Jaw, with Use of Plates in, by W. W. Allport, D.D.S. 514

Fractured Clavicle, a New Mode of Dressing, by J. W. Freer, M.D....517
Fever, Scarlet, Essay on, by Casper Morris, M.D....535
Fractures. Ununited, treated by Sub-cutaneous Perforation....421
Fracture of the Lower Jaw at its Neck, by M. O. Heydock, M.D....387
Febrile Movement in Disease, by J. R. Hayes, M.D....252

Glycerine in Phthisis, by N. S. Davis, M.D... 49
Graham, J. N., on Ulceration of Os Uteri...... 70
Glysters of Alum in Dysentery, by Dr. Hamon.170
Gutta Percha in Treatment of Fractures of Lower Jaw....514

Hydrocephalus, Chronic, treated by Injections of Iodine....600
Hernia, Strangulated, by Wm. Dickinson, M.D....497
Hepatic Abscess, Two Cases of....439
Heydock, M. O., on Fracture of Lower Jaw...387
Harper, J. Drake, on Pseudo-Membranous Laryngitis....300
Haines, W. C., on Peritoneal Inflammation.308
Hinkle, J. M., on Changes in the Treatment of Disease....311
Holmes, E. L., Translations by......318, 322, 224
Hatch, Ira, on Tully's Materia Medica....330
Hayes, J. R., on Febrile Movement....252
Hall, Mashall, Ready Method in Asphyxia...210
Hæmoptysis, Case of, by J. M. Kindall, M.D.214
Hæmatemesis, Acute, by B. F. Schenck, M.D., 153
Human Histology, by E. R. Peaslee, M.D...172
Holmes, E. L., on the Uses of the Ophthalmoscope....101
Hand Book of Practice and Memoranda....133
Henry Co. Medical Society, Meeting of....135
Honey Bees as a Diuretic....144
Homœopathy and the Chicago City Hospital. 50
Hymenial.... 50
Holmes, E. L., on the Human Eye....623

Illinois State Medical Society......475, 281, 240
Indiana State Medical Society....285
Inflammation, Case of Peritoneal....308
Inhalation of Nitrate of Silver, by W. H. Studly, M.D....309
Isham, Ralph N., on Puerperal Convulsions, 485
Inversio Uteri, by Dr. A. Fisher....536, 510
Inebriate Asylum for the State of New York, 544
In Memoriam....665

Janitor....240

Knee Joint, Resection of, by Daniel Brainard, M.D....293

Leucorrhœa in Children.... 36
Lower Extremities, Malformation of, by D. H. Adam....165
London Lancet....240
Laryngitis, or True Croup, by J. Drake Harper, M.D....300
Lallemand, M., on Spermatorrhœa....465
Lectures on the Principles and Practice of Medicine, by Watson....597

Medical Dep'nt of University of Michigan...601
Manual of Practical Medicine, by T. H. Tanner....533
McLean County Medical Society....469
Mercer County Medical Society....470
Medical Charities....477
Medical Journal Changes....478
Medical Colleges, Number of, in U. S. A......478
Muscular Exercise, Therapeutics of, etc......357
Medicines, Examine them....394
Measles a Second Time in the Same Patient, 304
Medical Education....306
Materia Medica and Therapeutics, by T. D. Mitchell, M.D....261
Milk-Sickness, by J. P. DeBruler, M.D....206
Malformation, by S. W. Wallace, M.D....215
Manual of Medical Diagnosis, by A. W. Barclay, M.D....172
Mixture for the Teeth.... 41

North-American Medico-Chirurgical Review.176
New Journals....336, 177
Nerves, Influence of, in Inflammation....224
Natrophagie, a Case of....396
New Hampshire Med. Society Transactions..533

Ophthalmoscope, Uses of, by E. L. Holmes, M.D....101
Obstetrics, Principles and Practice of, by H. Miller, M.D....132
Ovarian Dropsy, successfully treated, by J. H. Brower, M.D....212
Œsophagus, Foreign Body in, by J. W. Redden, M.D....447
Os and Cervix Uteri, Diseases of....451
Obituary....507, 477

Physicians' Visiting List, etc., for 1859....599
Plain Talk with the Readers of the Journal.387
Plates Illustrative of Wilson on Diseases of the Skin....384
Placenta Prævia, by J. P. Terrell, M.D....297
Palmer, A. B., on Sulph. Quinia....229
Prolapse of the Funis, by J. G. Taylor, M.D..227
Philadelphia Co. Medical Society....129
Polypiform Concretions, by P. A. Allaire, M.D.117
Prince, David, Case of Empyema.... 95
Prolific.... 49
Paoli, Gerhard, on Ipecacuanha in Delirium Tremens....576
Personal....662

Quarantine of New York....478

Roler, E. O. F., Clinical Cases....662
Resection of Elbow Joint and Ulna, by J. W. Freer, M.D....632
Read, E., an Address, etc.... 5
Rational Therapeutics, etc., a Prize Essay, by Worthington Hooker, M.D.... 84
Rock Island Co. Medical Society....329
Rush Medical College....543, 476, 414
Resection of Bones, by D. Brainard, M.D., etc.435
Resignations....477
Report on Practical Medicine, by F. K. Bailey, M. D....645, 581, 519
Reddy, John, on Compression and Perchloride of Iron Injection in Aneurism....598
Rheumatism....322

Sulphate of Copper in Croup....667
Strychnine Poisoning relieved by Camphor..668
Scabies, a Specific for....667
Spinal Meningitis, Acute, by R. C. Russ, M.D.164
Stimulation and Reaction, by O. Smith, M.D.549
Subscribers....536
Shelby Medical College....476
Studly, W. H., on Inhalation of Nit Silver.309
Spontaneous Mortification....324
Surgical Cases....157, 245

Trousseau's Opinion of Tracheotomy....666
Tabes Dorsalis, treated by Galvanic Currents.318
Tennessee State Medical Society, Transactions of....398

Valerianate of Ammonia in Epilepsy....170
Vagina, Occlusion of, by Ernst Schmidt, M.D....197

### RECEIPTS ON SUBSCRIPTIONS,

UP TO JANUARY 20TH, 1858.

### ILLINOIS.

| | | | |
|---|---|---|---|
| Dr. W. H. Hogan, | VOL. 6, | 1857, | $2 00 |
| " L. D. Smedley, | " 1, | 1858, | 2 00 |
| " J. A. Brenneman, | " 5&6, | 1856-7, | 4 00 |
| " S. W. Noble, | " 6, | 1857, | 2 00 |
| " J. L. Hoover, | " 1, | 1858, | 2 00 |
| " W. W. Alport, | " 1, | " | 2 00 |
| " H. Shoemaker, | " 6, | 1857, | 2 00 |
| Jonesboro Med. Soc'y, | " 6, | " | 2 00 |
| Dr. Wm. Bentley, | " 1, | 1858, | 2 00 |
| " J. F. Faris, | " 6, | 1857, | 2 00 |
| " B. E. Dodson, | " 5, | 1856, | 2 00 |
| " J. W. McKinney, | " 6, | 1857, | 2 00 |
| " T. D. Fisher, | " 1, | 1858, | 2 00 |
| " C. Goodbrake, | " 1, | " | 2 00 |
| " J Leeds, | " 6, | 1857, | 2 00 |
| " A. J. Crain, | " 6, | " | 2 00 |
| " G. M. Fox, | VOLS. 5, 6& 1, | 1856, 7&8, | 6 00 |
| " H. W. Hopkins, | VOLS. 6&1, | 1857-8, | 4 00 |
| " E. Y. Nichols, | " 1, | 1858, | 2 00 |
| " Orrin Peake, | " 1, | " | 2 00 |

### MICHIGAN.

| | | | |
|---|---|---|---|
| Dr. R. Stephenson, | VOLS. 5&6, | 1856-7, | $4 00 |

### INDIANA.

| | | | |
|---|---|---|---|
| Dr. S. H. Buell, | VOLS. 5&6, | 1856-7, | $5 00 |
| " R. D. Hutchinson, | " 6, | 1857, | 2 00 |
| " R. Q. Wilson, | " 1, | 1858, | 2 00 |
| " Sam'l Ross, | " 6, | 1857, | 2 00 |
| " Robert Winton, | " 1, | 1858, | 2 00 |
| " R. C. Russ, | " 6, | 1857, | 2 00 |
| " J. P. Tueker, | " 6&1, | 1857-8, | 4 00 |
| " A. Chapman, | " 1, | 1858, | 2 00 |

### WISCONSIN.

| | | | |
|---|---|---|---|
| Dr. O. D. Coleman, | VOL. 6, | 1857, | $2 00 |
| " J. H. Bartlett, | " 5&6, | 1856-7, | 2 00 |

### IOWA.

| | | | |
|---|---|---|---|
| Dr. W. H. Fletcher, | VOL. 1, | 1858, | $2 00 |

### KANSAS.

| | | | |
|---|---|---|---|
| Dr. W. Manson, | VOL. 1, | 1858, | $2 00 |

### CALIFORNIA.

| | | | |
|---|---|---|---|
| Dr. Ira E. Oatman, | VOL. 1, | 1858, | $2 00 |

